# Causal effects of autoimmune diseases on temporomandibular disorders and the mediating pathways: a Mendelian randomization study

**DOI:** 10.3389/fimmu.2024.1390516

**Published:** 2024-07-09

**Authors:** Xin Chen, Zheng Cheng, Junyu Xu, Qianyi Wang, Zhibai Zhao, Qianglin Jiang

**Affiliations:** ^1^ Department of Oral and Maxillofacial Surgery, Jiangyin People’s Hospital Affiliated to Nantong University, Jiangyin, China; ^2^ Department of Cardiology, Jiangyin People’s Hospital Affiliated to Nantong University, Jiangyin, China; ^3^ Department of Oral Mucosal Diseases, The Affiliated Stomatological Hospital of Nanjing Medical University, Nanjing, China

**Keywords:** autoimmune diseases, Mendelian randomization analysis, temporomandibular disorders, mediation analysis, rheumatoid arthritis, multiple sclerosis

## Abstract

**Background:**

The role of autoimmune diseases (ADs) in temporomandibular disorders (TMDs) has been emphasized in observational studies. However, whether the causation exists is unclear, and controversy remains about which specific disorder is destructive in TMDs. This Mendelian randomization (MR) study aims to estimate the causal effect of common ADs on TMDs.

**Methods:**

Genetic data from published genome-wide association studies for fourteen common ADs, specifically multiple sclerosis (MS, N = 15,283), ankylosing spondylitis (AS, N = 22,647), asthma (N = 408,422), celiac disease (N = 15,283), Graves’ disease (N = 458,620), Hashimoto thyroiditis (N = 395,640), primary biliary cirrhosis (PBC, N = 11,375), primary sclerosing cholangitis (PSC, N = 14,890), psoriasis vulgaris (N = 483,174), rheumatoid arthritis (RA, N = 417,256), systemic lupus erythematosus (SLE, N = 23,210), Type 1 diabetes (T1D, N = 520,580), inflammatory bowel disease (IBD, N = 34,652), and Sjogren’s syndrome (SS, N = 407,746) were collected. Additionally, the latest summary-level data for TMDs (N = 228,812) were extracted from the FinnGen database. The overall effects of each immune traits were assessed via inverse-variance weighted (IVW), weighted median, and MR-Egger methods, and performed extensive sensitivity analyses. Finally, 731 immune cell phenotypes (N = 3,757) were analyzed for their mediating role in the significant causality.

**Results:**

Univariable MR analyses revealed that genetically predicted RA (IVW OR: 1.12, 95% CI: 1.05-1.19, *p* < 0.001) and MS (IVW OR: 1.06, 95% CI: 1.03-1.10, *p* = 0.001) were associated with increased risk of TMDs. Two out of 731 immune cell phenotypes were identified as causal mediators in the associations of RA with TMDs, including “CD25++ CD8+ T cell % CD8+ T cell” (mediation proportion: 6.2%) and “CD3 on activated CD4 regulatory T cell” (5.4%). Additionally, “CD127 on granulocyte” mediated 10.6% of the total effect of MS on TMDs. No reverse directions, heterogeneity, and pleiotropy were detected in the analyses (*p* > 0.05).

**Conclusion:**

This MR study provides new evidence regarding the causal impact of genetic predisposition to RA or MS on the increased risk of TMDs, potentially mediated by the modulation of immune cells. These findings highlight the importance for clinicians to pay more attention to patients with RA or MS when consulting for temporomandibular discomfort. The mediating role of specific immune cells is proposed but needs further investigation.

## Introduction

Temporomandibular disorders (TMDs) are characterized by craniofacial pain involving the temporomandibular joints (TMJ), masticatory muscles, or surrounding tissues (1). Autoimmunity, as one of the multifaceted etiologies of TMDs, plays an important role in pain control, bone remolding, and the effectiveness of corticosteroids treatment (1, 2). While higher prevalence rates of TMDs have been observed in individuals with autoimmune diseases (ADs) (3–5), the causality and underlying mechanism remain unclear.

ADs primarily involve the immune system mistakenly attacking the body’s own tissues, leading to chronic inflammation that may contribute to TMDs (6). Numerous studies have reported elevated TMDs prevalence in patients with rheumatoid arthritis (RA), ankylosing spondylitis (AS), systemic lupus erythematosus (SLE), and multiple sclerosis (MS) compared with healthy individuals (7–9). Notably, robust evidence, including meta-analysis, imaging studies, and animal experiments, supports a strong association between RA and TMDs (6, 10, 11). The involvement of the TMJ in RA patient may follow similar destruction patterns as in other joints, such as synovial hyperemia, lymphocyte infiltration, bone degeneration, and fibrous adhesion (12). This condition can lead to various TMDs, presented with arthralgia, restricted jaw movements, morning stiffness, and joint noises. TMJ radiographs in RA patients may show abnormalities in cortical integrity, jaw asymmetry, and joint space narrowing (13). Additionally, immunotherapy has shown promise in alleviating TMDs symptoms in patients newly diagnosed with RA or Sjogren’s syndrome (SS) (6). Biologically, shared pathways between ADs and TMDs have been identified. For instance, TMDs patients exhibit elevated levels of regulated upon activation normal T cell expressed and secreted (RANTES) in TMJ synovial fluid, promoting macrophage migration, osteoclast formation, and bone resorption (14). Moreover, increased levels of cytokines such as IL-1β, IL-17, and IL-22, which are associated with joint pain and articular bone degeneration, have been observed in patients with TMDs (15). Serum levels of RANTES chemokine and inflammatory cytokines are also notably elevated in several ADs, including autoimmune Addision disease, Graves’ disease, and Pemphigus Vulgaris (16–18). Collectively, these studies suggest an immune-related mechanism linking ADs to the pathogenesis of TMDs. Conversely, TMDs-related pain and psychological distress may exacerbate autoimmune conditions (19, 20), indicating a bidirectional relationship. However, establishing causal relationships between specific ADs, immune dysregulation, and TMDs remains challenging due to limitations such as small sample sizes and residual confounding (3–5),

Mendelian randomization (MR) is a powerful method used to establish causal relationships by leveraging specific genetic variations directly associated with a particular exposure (21). During fertilization, the random distribution of genetic variants mirrors the principles of randomized controlled trials (RCTs), effectively reducing the likelihood of bias in causal inferences, including confounding factors like age and sex (21). Furthermore, since genotype formation precedes the onset of diseases and is generally unaffected by disease progression, the potential for reverse causality is significantly minimized (21). Additionally, multivariable MR (MVMR) has emerged as a pivotal tool for estimating the complex pathways through which an exposure influences the outcome (22). Given the limited understanding of the mechanisms underlying ADs and TMDs, recognizing the influence of specific immune cell phenotypes is particularly crucial.

The availability of the latest genome-wide data for common ADs, numerous immunophenotypes, and TMDs has provided robust genetic instruments for MR analysis (23–25), effectively addressing concerns regarding weak-instrument bias. Recent MR investigations have consistently supported the causal link between specific ADs and conditions such as osteoporosis, multisite chronic pain, and Alzheimer’s disease (25–27). However, to date, no MR studies have specifically focused on the causal effects of ADs on TMDs. Thus, our primary aim was to examine the causal effects of 14 common ADs on TMDs using comprehensive MR analysis. Additionally, the potential mediating role of immune cells was explored in our observed causality to gain deeper insights into the underlying mechanisms.

## Materials and methods

### Study design

**Figure 1:**
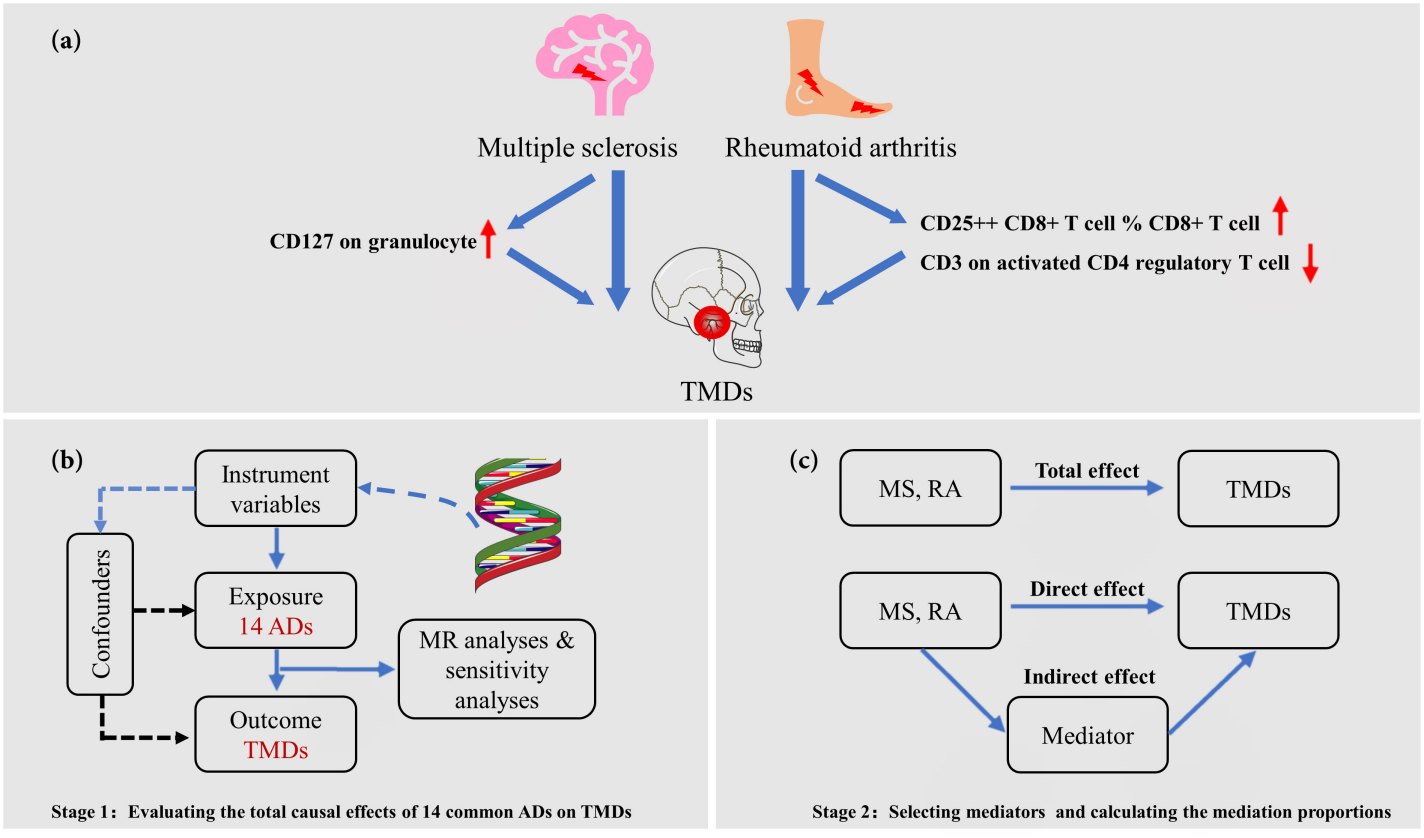
Overview of the present MR study. **(A)** Overview of this study, which comprises two stages of Mendelian randomization (MR) analyses; **(B)** The first stage involves univariable MR (UVMR) to investigate the causal effects of 14 common autoimmune diseases (ADs) on temporomandibular disorders (TMDs). Genetically predicted rheumatoid arthritis (RA) and multiple sclerosis (MS) have a causal effect on the onset of TMDs; **(C)** In the second stage, we utilized a two-step MR approach to identify potential causal mediators, specifically 731 immune cell phenotypes, in the associations between selected ADs and TMDs.

An overview of our study design is shown in **Figure 1** (designed by X. Chen and Q. Jiang). The genetic variants must satisfy the following three assumptions (28): First, genetic variants are directly related to exposure. Second, genetic variants are independent of any confounding variables. Third, genetic variants influence outcomes only through exposure. This MR analysis strictly follows the Strengthening Epidemiological Observation Research Report guidelines (29). As this MR study was based on the previously collected and published data, no ethics approval was required.

### Data resources

The targeted ADs include MS, AS, asthma, celiac disease, Graves’ disease, Hashimoto thyroiditis, primary biliary cirrhosis (PBC), primary sclerosing cholangitis (PSC), psoriasis vulgaris, RA, SLE, Type 1 diabetes (T1D), inflammatory bowel disease (IBD), and SS. Specifically, genetic instrumental variables (IVs) for asthma (56,167 cases, 352,255 controls) and SS (372 cases, 407,374 controls) were obtained from genome-wide association studies (GWAS) in the UK Biobank (30, 31), a large prospective study with over 500,000 participants aged 40-69 years. Additionally, genetic IVs for MS (4,888 cases, 10,395 controls), AS (9,069 cases, 13,578 controls), celiac disease (4,533 cases, 10,750 controls), Grave’s disease (1,678 cases, 456,942 controls), Hashimoto thyroiditis (15,654 cases, 379,986 controls), psoriasis vulgaris (5,072 cases, 478,102 controls), RA (8,255 cases, 409,001 controls), SLE (7,219 cases, 15,991 controls), T1D (18,942 cases, 501,638 controls), and IBD (12,882 cases, 21,770 controls) were sourced from the largest GWAS meta-analyses for each respective disease (32–38). Summary statistics of PBC were derived from the latest GWAS, including 2,861 cases from UK PBC Consortium and 8,514 controls from British Birth Cohort and National Blood Service (39). Genetic data of PSC were gathered from the International PSC Study Group (2,871 cases, 12,019 controls) (40). Detailed diagnostic criteria for each disease can be found in the original publications.

FinnGen, a large public-private partnership, has collected and analyzed genome and health data from 500,000 Finnish biobank donors to understand the genetic basis of diseases. The latest dataset release for TMDs is from December 2023 (R10 version). The involved conditions are mainly intra-articular TMDs, such as Costen complex or syndrome, derangement of TMJ, snapping jaw, and temporomandibular joint-pain-dysfunction syndrome (https://risteys.finngen.fi/endpoints/TEMPOROMANDIB). Individuals with jaw dislocation, jaw sprain and strain, and pain in limb, back, neck, and head were excluded. A total of 6,314 cases of TMDs and 222,498 controls were acquired from the GWAS data for this investigation. There was no sample overlap between ADs and TMDs in this MR study.

A total of 731 immunophenotypes, including absolute cell counts (n = 118), median fluorescence intensities reflecting (MFI) surface antigen level (n = 389), morphological parameters (n = 32), and relative cell counts (n = 192) were included (N = 3,757) (23). Specifically, MFI and relative counts comprised a range of immune cells, such as B cells, dendritic cells, monocytes, myeloid cells, TBNK, and Treg panels. All GWAS analyses in this study were exclusively conducted on populations of European descent, ensuring that the necessary ethical approvals and participant consents were diligently obtained. The details of the research period, research consortium, sample size, and data source are listed in **Table 1**. Data was collected by Z. Cheng and Z. Zhao.

**Table 1:** Details of the GWAS included in the Mendelian randomization.

Phenotypes	Research institution/ conductor	Research location	Research period	Sample size Total (cases/controls)	Ancestry	Data type	Data source
Exposure							
AS	IGAS (33)	Australia	NA	22,647 (9,069/13,578)	European	Binary	https://gwas.mrcieu.ac.uk/datasets/ebi-a-GCST005529/
Asthma	UK Biobank (30)	United Kingdom	2006-2010	408,422 (56,167/352,255)	European	Binary	https://gwas.mrcieu.ac.uk/datasets/ebi-a-GCST90014325/
MS	Andlauer et al. (32)	Germany	2009-2016	15,283 (4,888/10,395)	European	Binary	https://gwas.mrcieu.ac.uk/datasets/ebi-a-GCST003566/
Celiac disease	Dubois et al. (34)	United Kingdom	2007-2010	15,283 (4,533/10,750)	European	Binary	https://gwas.mrcieu.ac.uk/datasets/ebi-a-GCST000612/
Graves’ disease	Sakaue et al. (35)	United Kingdom & Finland	2006-2020	458,620 (1,678/456,942)	European	Binary	https://gwas.mrcieu.ac.uk/datasets/ebi-a-GCST90018847/
Hashimoto thyroiditis	Sakaue et al. (35)	United Kingdom & Finland	2006-2020	395,640 (15,654/379,986)	European	Binary	https://gwas.mrcieu.ac.uk/datasets/ebi-a-GCST90018855/
PBC	UK-PBC Consortium (39)	United Kingdom	2007-2012	11,375 (2,861/8,514)	European	Binary	https://gwas.mrcieu.ac.uk/datasets/ebi-a-GCST005581/
PSC	IPSCSG (40)	United Kingdom & Scandinavia	2010-2017	14,890 (2,871/12,019)	European	Binary	https://gwas.mrcieu.ac.uk/datasets/ieu-a-1112/
Psoriasis vulgaris	Sakaue et al. (35)	United Kingdom & Finland	2006-2020	483,174 (5,072/478,102)	European	Binary	https://gwas.mrcieu.ac.uk/datasets/ebi-a-GCST90018907/
RA	Sakaue et al. (35)	United Kingdom & Finland	2006-2020	417,256 (8,255/409,001)	European	Binary	https://gwas.mrcieu.ac.uk/datasets/ebi-a-GCST90018910/
SLE	Bentham et al. (36)	Spain & Italy & Turkey & America	NA	23,210 (7,219/15,991)	European	Binary	https://gwas.mrcieu.ac.uk/datasets/ebi-a-GCST003156/
T1D	Chiou et al. (37)	United Kingdom & Finland & Ireland & America	2001-2020	520,580 (18,942/501,638)	European	Binary	https://gwas.mrcieu.ac.uk/datasets/ebi-a-GCST90014023/
IBD	IIBDGC (38)	Norway & Sweden & Denmark & Spain & Italy &Canada & America & Slovenia & Lithuania & Germany & Belgium & Netherlands & United Kingdom & Australia & New Zealand	NA	34,652 (12,882/21,770)	European	Binary	https://gwas.mrcieu.ac.uk/datasets/ieu-a-31/
SS	UK Biobank (31)	United Kingdom	2006-2015	407,746 (372/407,374)	European	Binary	https://gwas.mrcieu.ac.uk/datasets/ebi-a-GCST90013879/
Outcome							
TMDs	FinnGen	Finland	2017-2023	228,812 (6,314/222,498)	European	Binary	https://storage.googleapis.com/finngen-public-data-r10/summary_stats/finngen_R10_TEMPOROMANDIB.gz
Mediator							
Immune cells	Orru et al. (23)	Italy	2001-2020	3,757	European	Continuous	https://gwas.mrcieu.ac.uk/datasets/ebi-a-GCST90001391/ - https://gwas.mrcieu.ac.uk/datasets/ebi-a-GCST90002121/

AS, ankylosing spondylitis; MS, multiple sclerosis; PBC, primary biliary cirrhosis; PSC, primary sclerosing cholangitis; RA, rheumatoid arthritis; SLE, systemic lupus erythematosus; T1D, Type 1 diabetes; IBD, inflammatory bowel disease; SS, Sjogren’s syndrome; TMDs, temporomandibular disorders; IGAS, International Genetics of Ankylosing Spondylitis Consortium; IPSCSG, International PSC Study Group; IIBDGC, International Inflammatory Bowel Disease Genetics Consortium; NA, not available.

### Selection of genetic instruments

For selecting single nucleotide polymorphisms (SNPs) in an MR study, the following criteria are typically adopted to ensure robust and reliable causal inferences (24, 25). Firstly, SNPs are chosen based on their association with the exposure of interest at a genome-wide significance level, typically *p* < 5×10^-8^, to reduce the likelihood of false-positive associations. Additionally, SNPs must be independent of each other to avoid confounding due to linkage disequilibrium (LD). This could be achieved through the LD clumping procedure (r^2^ ≥ 0.001, clumping window ≤ 10,000 kb), where the SNP with the strongest association remains. The selected SNPs were then uploaded to the PhenoScanner V2 website (http://www.phenoscanner.medschl.cam.ac.uk) to eliminate those associated with potential confounders (smoking behavior, alcohol consumption, painful and psychosocial conditions) and the outcome (24). Furthermore, the *F-*statistics were calculated to assess the instrumental strength, with *F*-statistics >10 set as the threshold of strong IVs. SNPs with *F*-statistics < 10 were excluded.

### Statistical analysis

The inverse variance weighted (IVW) method, the most efficient analysis method when all genetic variants are valid IVs, was selected *a priori* to estimate the causal effects (26). Given the number of tests performed, the statistical significance level was adjusted to 0.003 (0.05/14) to define the evidence of a causal effect.

Furthermore, a two-step MR method was adopted to investigate the mediating effect of an intermediate risk factor in the causal associations between ADs and TMDs. Briefly, the IVW method was used to estimate the causal effects of selected autoimmune trait on 731 immune cell phenotypes, and reverse analysis was also performed to rule out the presentation of bidirectionality. Following that, multivariable IVW (MV-IVW) was applied to estimate the causal effect of the mediator on TMDs, adjusting for the corresponding immune disorder. The association between the ADs and the mediator, as well as the association between the mediator and TMDs, should be in the same direction. Finally, the Delta method was utilized to obtain the mediation proportion of each mediator and the 95% confidence intervals (41).

### MR sensitivity analysis

Several sensitivity analyses, such as MR Egger, weighted median, and Mendelian randomized polymorphism RESidual Sum and Outlier (MR-PRESSO) method, were conducted to validate the robustness of the IVW results in the UVMR analysis (24, 25). If any outlier SNPs identified by MR-PRESSO, the MR analysis was restarted after removing outliers. Additionally, MVMR-Egger, MVMR-median, and MVMR-Lasso methods were applied to validate the robustness of the MV-IVW results in the mediation analysis. The third assumption, generally referred to as no pleiotropy, could be tested indirectly using various statistical methods (24). Consequently, Cochran’s Q test was performed to assess heterogeneity among different genetic variations, and the MR-Egger intercept test was used for horizontal pleiotropy (27). Finally, a leave-one-out analysis (LOO) was conducted to evaluate whether the stability of the results was affected by a single SNP.

All MR analyses were performed by Q. Wang and J. Xu, using R software (version 4.3.0) through the TwoSampleMR package (version 0.5.6), MRPRESSO (version 1.0), MendelianRandomization (version 0.7.0) and MVMR (version 0.4.0).

## Results

### Instrumental variables selection

Detailed information on the SNPs associated with ADs, including comprehensive characteristics after initial screening, removal of confounding factors (such as smoking, pain, anxiety, tension, depression, worry or nerve-related conditions), and data harmonization, can be accessed in **Supplementary Tables 1-3**. A total of 19 SNPs for RA, 38 SNPs for SLE, 19 SNPs for PBC, 9 SNPs for Hashimoto thyroiditis, 11 SNPs for celiac disease, 57 SNPs for asthma, 68 SNPs for T1D, 49 SNPs for IBD, 21 SNPs for Graves’ disease, 13 SNPs for PSC, 21 SNPs for MS, 7 SNPs for SS, 9 SNPs for psoriasis vulgaris, and 22 SNPs for AS were adopted. The minimum calculated *F-*statistic was 27.3 (**Supplementary Table 3**), ensuring the instrumental strength.

### Causal effects of ADs on TMDs

**Figure 2:**
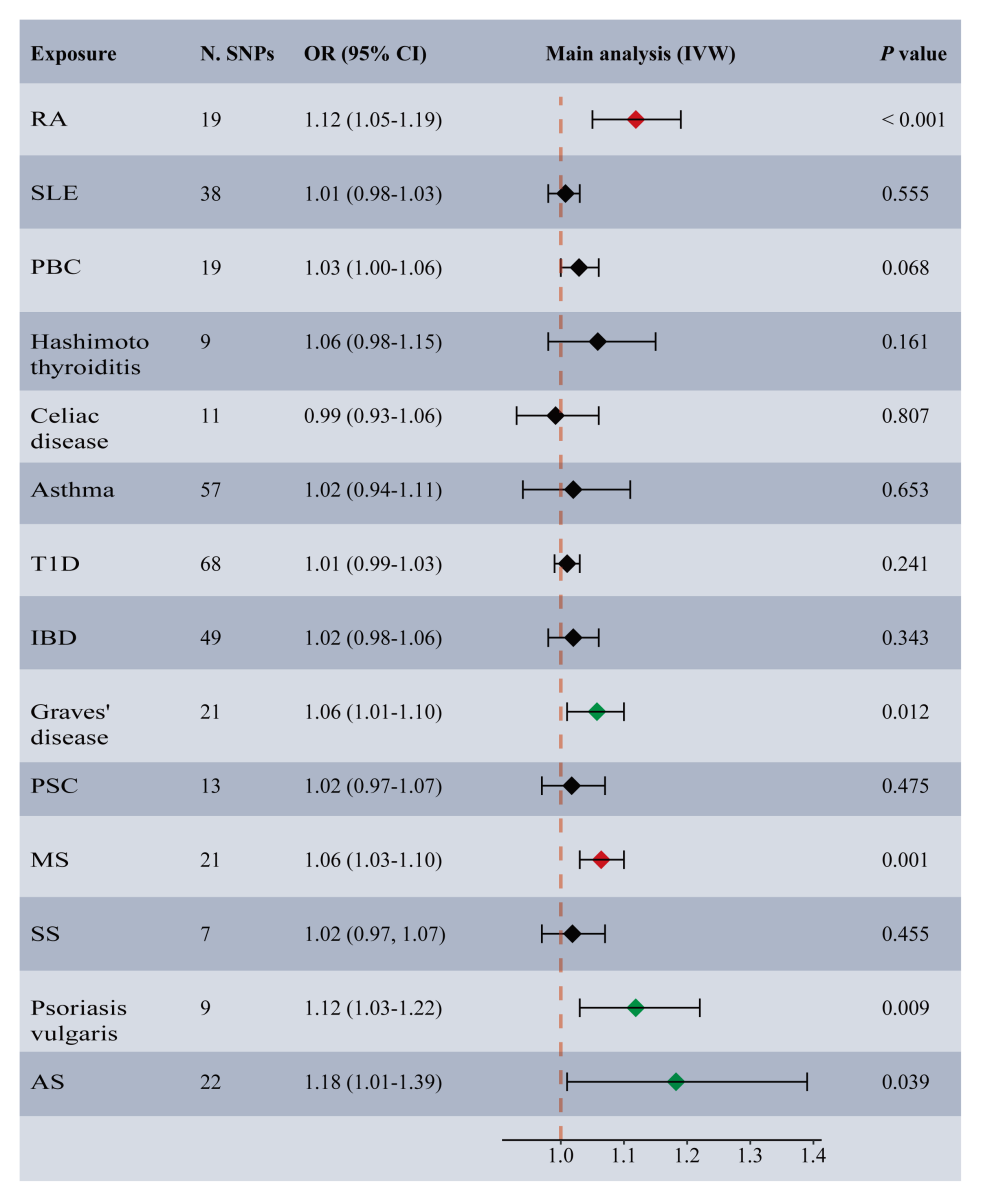
Forest plot depicting MR results for the association of genetically proxied autoimmune diseases with temporomandibular disorders. N. SNPs, number of SNPs used in MR; OR, odds ratio; CI, confidence intervals; IVW, inverse variance weighted; AS, ankylosing spondylitis; MS, multiple sclerosis; PBC, primary biliary cirrhosis; PSC, primary sclerosing cholangitis; RA, rheumatoid arthritis; SLE, systemic lupus erythematosus; T1D, Type 1 diabetes; IBD, inflammatory bowel disease; SS, Sjogren’s syndrome; the red diamond indicating significant causal effects; the green diamond indicating suggestive causal effects.

**Figure 3:**
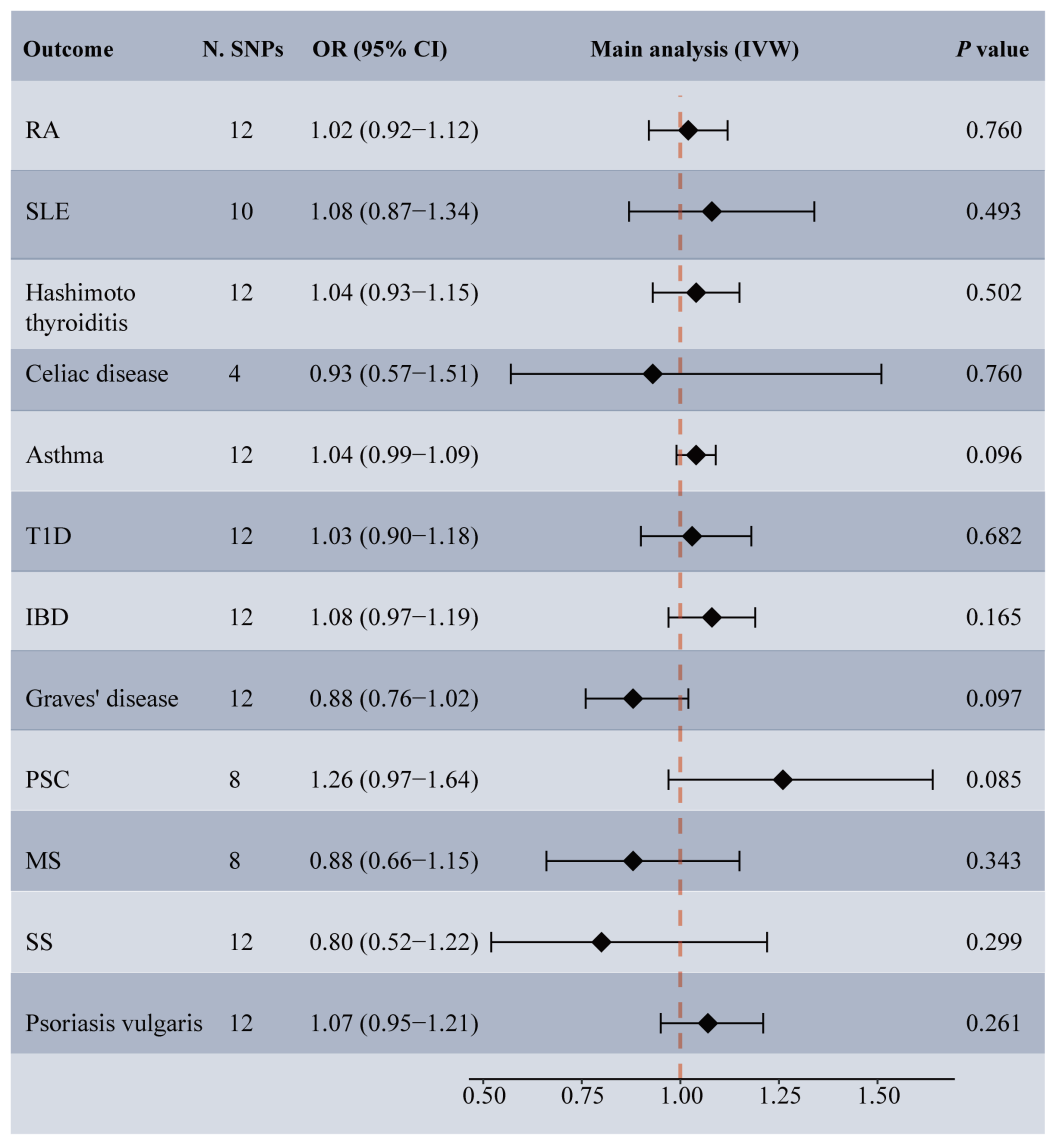
Forest plot depicting MR results for the association of genetically proxied temporomandibular disorders with autoimmune diseases. N. SNPs, number of SNPs used in MR; OR, odds ratio; CI, confidence intervals; IVW, inverse variance weighted; MS, multiple sclerosis; PSC, primary sclerosing cholangitis; RA, rheumatoid arthritis; SLE, systemic lupus erythematosus; T1D, Type 1 diabetes; IBD, inflammatory bowel disease; SS, Sjogren’s syndrome.

Among the 14 common autoimmune phenotypes, genetically predicted RA (IVW OR: 1.12, 95% CI: 1.05-1.19, *p* < 0.001) and MS (IVW OR: 1.06, 95% CI: 1.03-1.10, *p* = 0.001) demonstrated a causal effect on the onset of TMDs (**Figure 2**). Consistent estimates were obtained through three other MR methods (**Supplementary Table 4**). Furthermore, a suggestive association of Grave’s disease (IVW OR: 1.06, 95% CI: 1.01-1.10, *p* = 0.012), psoriasis vulgaris (IVW OR: 1.12, 95% CI: 1.03-1.22, *p* = 0.009), or AS (IVW OR: 1.18, 95% CI: 1.01-1.39, *p* = 0.039) with the risk of TMDs was observed. No evidence of horizontal pleiotropy (all intercept *p* > 0.05), heterogeneity, or any outlier was found throughout the analyses (**Table 2**; **Supplementary Figures 1, 2**). LOO analyses confirmed that no specific IVs significantly influenced causal inferences (**Supplementary Figure 3**). Notably, reverse MR indicated that genetically predicted TMDs had no causality on each autoimmune trait (**Figure 3**; **Supplementary Table 5**).

**Table 2:** Sensitivity analysis for the associations between autoimmune diseases and temporomandibular disorders.

Exposure	Cochran Q test			MR-Egger		MR-PRESSO
	Q value	*I^2^*	*p*	Intercept	*p*	Global test. *p*
AS	15.097	0.391	0.818	0.000	0.990	0.822
Asthma	59.866	0.065	0.337	0.002	0.815	0.413
MS	27.243	0.266	0.129	-0.001	0.916	0.179
Celiac disease	13.259	0.246	0.210	-0.009	0.765	0.200
Graves’ disease	17.978	0.112	0.589	-0.010	0.496	0.650
Hashimoto thyroiditis	8.930	0.104	0.348	-0.011	0.576	0.197
PBC	21.591	0.166	0.251	0.015	0.162	0.264
PSC	11.947	0.004	0.450	0.010	0.542	0.472
Psoriasis vulgaris	10.233	0.218	0.249	0.003	0.902	0.124
RA	23.809	0.244	0.161	0.015	0.109	0.172
SLE	42.990	0.139	0.230	-0.003	0.794	0.277
T1D	77.600	0.137	0.177	-0.001	0.857	0.190
IBD	51.961	0.076	0.322	0.005	0.587	0.487
SS	9.987	0.399	0.125	-0.027	0.502	0.171

AS, ankylosing spondylitis; MS, multiple sclerosis; PBC, primary biliary cirrhosis; PSC, primary sclerosing cholangitis; RA, rheumatoid arthritis; SLE, systemic lupus erythematosus; T1D, Type 1 diabetes; IBD, inflammatory bowel disease; SS, Sjogren’s syndrome; MR-PRESSO, Mendelian randomized polymorphism RESidual Sum and Outlier.

### Mediating effects of immune cells in the associations of RA, MS with TMDs

**Figure 4:**
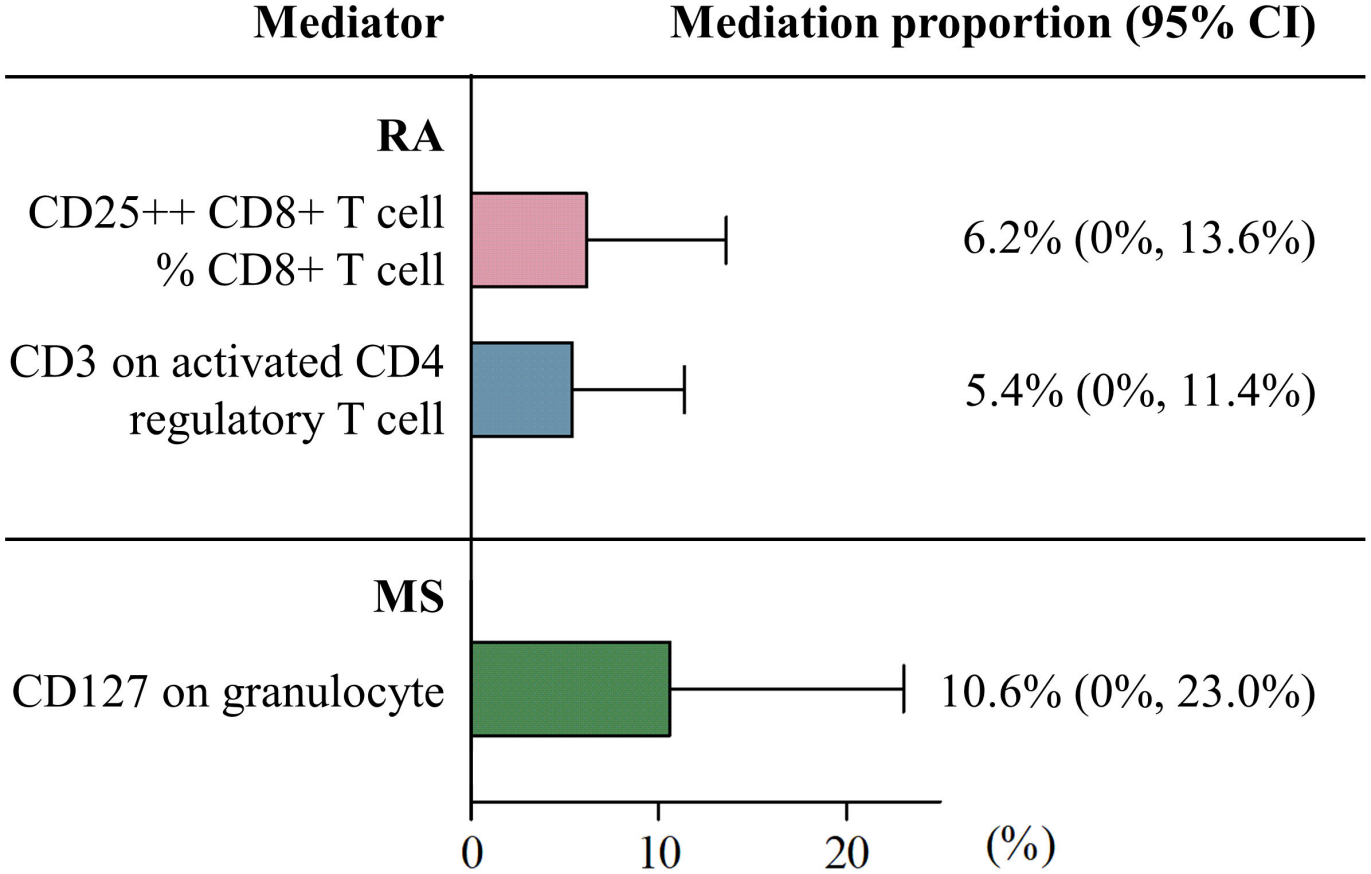
Two-step MR estimating proportions mediated by mediators in the causal associations of MS or RA with temporomandibular disorders. Histograms (bars) represent the mediation proportions (95% CI). For rheumatoid arthritis (RA), pink plots represent the proportions mediated by CD25++ CD8+ T cell % CD8+ T cell and blue plots represent the proportion mediated by CD3 on activated CD4 regulatory T cell; for multiple sclerosis (MS), green plots represent the proportion mediated by CD127 on granulocyte. CI, confidence interval.

Among the 731 candidate mediators, a total of three immune cell phenotypes met the criteria and were included in the calculation of mediation proportions. Specifically, RA was causally linked to a decreased level of CD3 on activated CD4 regulatory T cell (IVW beta: -0.10, 95% CI: -0.18, -0.01, *p* = 0.021) and increased CD25++ CD8+ T cell % CD8+ T cell (IVW beta: 0.11, 95% CI: 0.03-0.19, *p* = 0.006), while genetically predicted MS was significantly associated with higher expression of CD127 on granulocyte (IVW beta: 0.11, 95% CI: 0.04-0.17, *p* = 0.002) (**Supplementary Table 6**). No reverse relationships were observed among these variables (**Supplementary Table 6**). In the MVMR analyses, both CD3 on activated CD4 regulatory T cell (MV-IVW OR: 0.94, 95% CI: 0.90-0.98, *p* = 0.002) and CD25++ CD8+ T cell % CD8+ T cell (MV-IVW OR: 1.07, 95% CI: 1.01-1.13, *p* = 0.029) demonstrated evidence of a causal association with TMDs even after adjusting for RA (**Supplementary Table 7**). Similarly, CD127 on granulocyte had a causal effect on the risk of TMDs after adjusting for MS (MV-IVW OR: 1.06, 95% CI: 1.00-1.13, *p* = 0.043). Consistent associations were found across various sensitivity analysis approaches (**Supplementary Table 7**). Ranked by mediation proportion, CD127 on granulocyte mediated 10.6% of the total effect of MS on TMDs, followed by CD25++ CD8+ T cell % CD8+ T cell mediating the causal effect of RA on TMDs (6.2%), and CD3 on activated CD4 regulatory T cell (5.4%) (**Figure 4**).

## Discussion

The association between immunity and the risk of TMDs has garnered increasing interest within the scientific community. To the best of our knowledge, this is the first study to systematically investigate potential causal relationships between ADs and TMDs using MR approaches. Our findings suggest that genetically predicted RA and MS exert a significant causal effect on TMDs risk, while genetic susceptibility to Grave’s disease, psoriasis vulgaris, and AS shows a suggestive association with elevated TMDs risk. Furthermore, employing MVMR incorporating immune cells revealed that the influence of RA and MS on TMDs was partially mediated by certain relative cell counts and antigen levels.

Research into the relationship between immune traits and TMDs has a history spanning 70 years (6). The prevailing theory posits that the inflammatory response and alterations in cellular homeostasis may contribute to TMDs development (6). However, evidence linking ADs to TMDs risk remains inconclusive due to a dearth of high-quality RCTs and inconsistent findings in observational epidemiological research (3, 4). RA, a chronic autoimmune disease characterized by synovial joint inflammation, has been linked to TMJ pain prevalence ranging widely from 8% to 70% in RA patients (7, 9). Another observational study concluded that TMJ symptoms generally occurred within 5 years following the onset of RA (42). A recent systematic review consolidating data from 34 observational studies established RA as a significant risk factor for developing TMDs (6). However, Sem et al. reported a reduction in TMDs symptoms prevalence in newly diagnosed RA patients over a 3-year follow-up, particularly in the initial years (43). This discrepancy could be attributed to the efficacy of tailored immunosuppressive treatments in alleviating inflammation and enhancing various TMJ-associated outcomes (6).

Our study contributes significantly to existing literature in two key areas. Firstly, a causal link between RA and TMDs occurrence was established utilizing large-scale genetic data. Secondly, the intermediary roles played by specific immune cell markers in this association were observed. While B cells play a pivotal role in RA pathogenesis by producing autoantibodies, studies in collagen antibody-induced arthritis mice revealed no significant changes in B cell numbers within the TMJ compared to control mice (11). However, a notable increase in T cells was observed in TMJ when mechanical stress was applied to the mandibular condyle, suggesting their potential involvement in RA-associated TMDs (11). Our results suggest that the impact of RA on TMDs may be mediated through reduced CD3 expression on CD4 T cells in serum and an elevated percentage of CD25++ CD8+ T cells. These findings align with observational studies on TMDs patients, where chondrocytes and synovial fibroblasts secrete RANTES in early TMDs stages, promoting the migration of T cells and macrophages to the joints (14). IL-2, primarily derived from CD4 T cells and serving as the main CD25 ligand, could regulate T cell proliferation bidirectionally (44). Observational evidence indicated elevated levels of IL-2 in TMJ capsule fluid of TMDs patients (44) and an increased number of peripheral CD8+ CD25+ T cells in RA patients (45), suggesting a potential role for the IL-2 pathway in mediating RA-associated TMDs. Theoretically, heightened intercepts between CD25 and IL-2 could suppress proliferation and initiate apoptosis of T cells (46). Although the specific contribution of T cells to TMJ pathology remains unclear, our findings underscore the significance of functional imbalances in CD4 and CD8 T cells in RA-related TMDs.

Multiple sclerosis (MS) is characterized by immune-mediated attacks, primarily by T-helper lymphocytes Types 1 and 17, on the nerve fiber myelin sheath, leading to demyelination (47). Despite ongoing debates, recent meta-analysis findings by Minervini et al. suggest a significantly higher risk of TMDs in MS patients compared to healthy controls (RR: 2.10; 95% CI: 1.21-3.65) (5). However, the limited sample sizes (≤ 120 individuals), lack of follow-up, and various diagnostic criteria in these studies necessitate further validation (5). Our MR study provides genetic evidence supporting a causal link between MS and TMDs. The IL-7R comprises α chain (CD127) and γ chain subunits, the latter sharing receptors with interleukins such as IL-2, IL-4, IL-9, and IL-15 (48). Notably, IL-7Rα’s association ranks second only to major histocompatibility complex polymorphisms in MS risk (47). *In vivo* studies have demonstrated that downregulation of IL-7R induces apoptosis in oligodendrocytes via the JAK/STAT pathway (49). Moreover, elevated levels of inflammatory factors like IL-6, IL-7, and IL-8 have been observed in the masseter muscles of TMD patients, particularly during teeth clenching (50). These findings suggest the involvement of the IL7/IL-7R pathway in both MS and TMDs development, potentially contributing to their associations. However, the impact of IL-7 on neutrophils remains uncertain due to their minimal expression of IL-7R α chain, implying negligible effects on their chemotactic and phagocytic functions (51). Our mediation MR analysis indicates that MS may modulate the expression of certain immune cell surface molecules, such as increased CD127 on granulocytes, possibly activating the neutrophil IL-7/IL-7R pathway and exacerbating the inflammatory response around the TMJ. Additionally, MS patients commonly encounter symptoms of central nervous system sensitivity (52), which could serve as significant triggers for TMDs occurrence.

While some clinical studies have suggested a close association between immune disorders like SLE, SS, and Hashimoto thyroiditis and the prevalence of TMDs (4, 9, 53), this MR analysis did not find evidence supporting a causal link between these three immune traits and TMDs. Furthermore, the potential impacts of various gastrointestinal immune conditions were explored, with suggestive causality between PBC and TMDs. The weighted-median method provides reliable estimates of causal effects when fewer than half of the SNPs are invalid, whereas the MR-Egger method can yield robust inferences even when all instruments are invalid (24). In this MR study, all complementary methodologies produced consistent results and indicated no signs of heterogeneity or horizontal pleiotropy, underscoring the strength and reliability of our findings.

Effective management of both TMDs and ADs requires a multidisciplinary approach (1). Our research offers valuable insights into the prevention and treatment of TMDs, particularly by identifying adults with RA or MS as a high-risk population. Individuals with autoimmune conditions, already facing mobility or dexterity challenges, may further experience compromised oral function due to TMDs symptoms. Hence, regular follow-ups for early TMDs detection and timely intervention in RA or MS patients are crucial. Furthermore, a comprehensive evaluation of patients seeking dental care revealed concerning findings. Among 1,458 participants diagnosed with immune-mediated rheumatic diseases, 58% had not been informed about the oral risks associated with their immune condition by their dentist (54). Understanding how their immune disorder can impact TMDs risk may empower patients to actively participate in their healthcare, adhere to treatment plans, and seek appropriate interventions when experiencing symptoms related to jaw dysfunction. Additionally, it is essential that dentists stay updated on the medications their patient with RA or MS are receiving, along with possible side effects and interactions with oral health. Moreover, studies have suggested that concurrent treatment of immune diseases may alleviate temporomandibular pain and dysfunction (6). Therefore, collaborative efforts among dentists, immunologists, and other healthcare professionals are essential to provide holistic care, addressing the intricate relationship between immune diseases and the development of TMDs.

However, it is essential to exercise caution when interpreting our study findings due to several limitations. Firstly, the GWAS populations analyzed were of European descent, potentially limiting generalizability to other ethnicities. Secondly, inadequate control over confounding variables could introduce bias or inaccuracies into the results. Despite adjusting for some confounders in the UVMR analysis, others like immunosuppressant and nonsteroidal anti-inflammatory drug usage may have been overlooked. For instance, patients with SLE are particularly vulnerable to TMJ complications due to frequent glucocorticoid use (55). Thirdly, TMDs were globally categorized in available GWAS, and the specific causal relationship between immune disorders and specific TMDs subtypes remains unclear.

This MR study proposes hypotheses rather than definitive conclusions. Therefore, it is necessary to conduct large-scale, multicenter longitudinal cohort studies in the future to monitor the development and progression of TMDs in patients with RA or MS, identifying early biomarkers and predictive factors for TMDs. Additionally, the specific roles of CD4 regulatory T cells and granulocytes in the pathogenesis of TMDs deserve further research using techniques such as single-cell RNA sequencing and flow cytometry.

## Conclusion

Our MR estimates present compelling evidence regarding the causal impact of genetic predisposition to RA or MS on the increased risk of TMDs, potentially mediated by the modulation of immune cells. These findings offer novel insights into the underlying mechanisms linking immunity and TMDs, highlighting the importance for clinicians to pay more attention to patients with RA or MS when consulting for temporomandibular discomfort.

## Data availability statement

The original contributions presented in the study are included in the article/**Supplementary Material**. Further inquiries can be directed to the corresponding authors.

## Ethics statement

Ethics approval for the study involving humans was not required in accordance with the local and institutional requirements because the MR study was based on previously collected and published data

## Author contributions

XC: Conceptualization, Writing – original draft. ZC: Data curation, Resources, Software, Writing – review & editing. JX: Formal Analysis, Methodology, Writing – review & editing. QW: Validation, Writing – original draft, Writing – review & editing. ZZ: Resources, Software, Writing – review & editing. QJ: Conceptualization, Writing – review & editing.
