# Supplementary material for: Causal effects of autoimmune diseases on temporomandibular disorders and the mediating pathways: a Mendelian randomization study
Source: Front Immunol. 2024 Jul 9;15:1390516. doi: 10.3389/fimmu.2024.1390516 (PMC11263080; doi:10.3389/fimmu.2024.1390516)
Supplement: Supplementary file 1 [file DataSheet_1.docx]

**Supplementary figures:**

**Supplementary Fig. 1 Scatter plots of the causal relationship between 14 autoimmune diseases and temporomandibular disorders using different MR methods.**

**
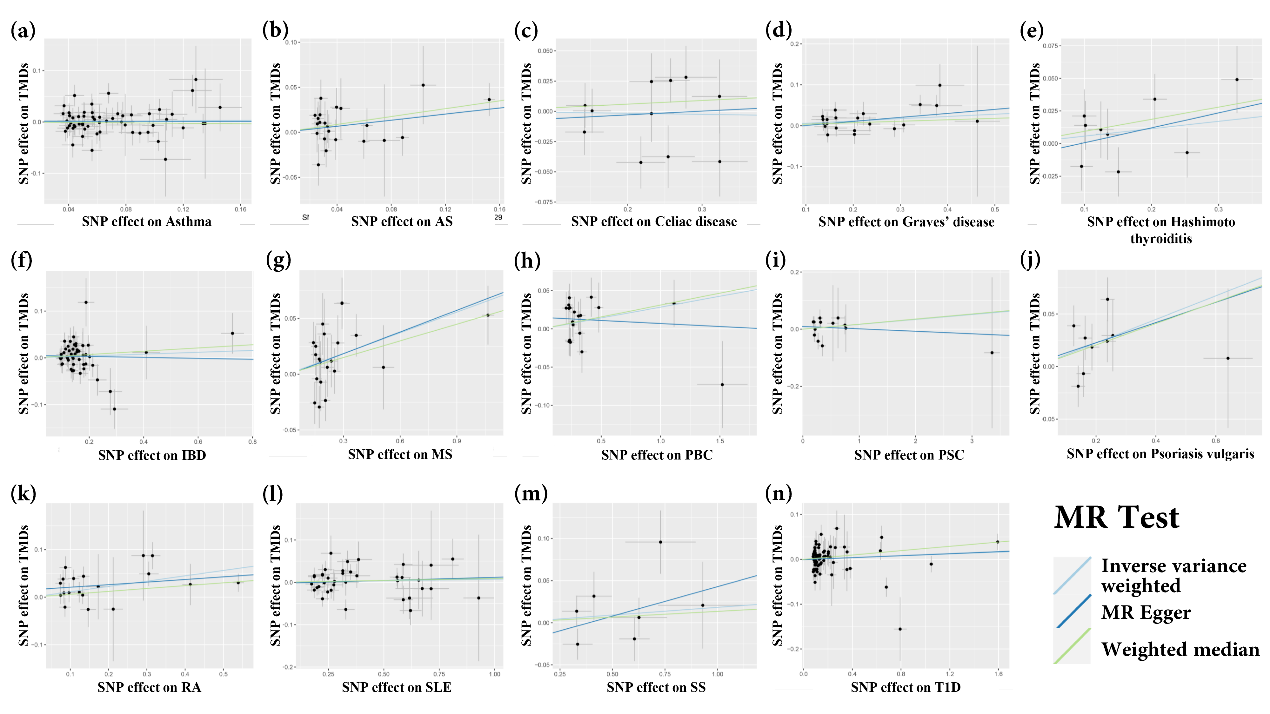
**

a. Causal estimates for asthma on temporomandibular disorders (TMDs); b. Causal estimates for ankylosing spondylitis (AS) on TMDs; c. Causal estimates for celiac disease on TMDs; d. Causal estimates for Graves’ disease on TMDs; e. Causal estimates for Hashimoto thyroiditis on TMDs; f. Causal estimates for inflammatory bowel disease (IBD) on TMDs; g. Causal estimates for multiple sclerosis (MS) on TMDs; h. Causal estimates for primary biliary cirrhosis (PBC) on TMDs; i. Causal estimates for primary sclerosing cholangitis (PSC) on TMDs; j. Causal estimates for psoriasis vulgaris on TMDs; k. Causal estimates for rheumatoid arthritis (RA) on TMDs; l. Causal estimates for systemic lupus erythematosus (SLE) on TMDs; m. Causal estimates for Sjogren’s syndrome (SS) on TMDs; n. Causal estimates for Type 1 diabetes (T1D) on TMDs.

**Supplementary Fig. 2 Funnel plots for IVW and MR-Egger method.**


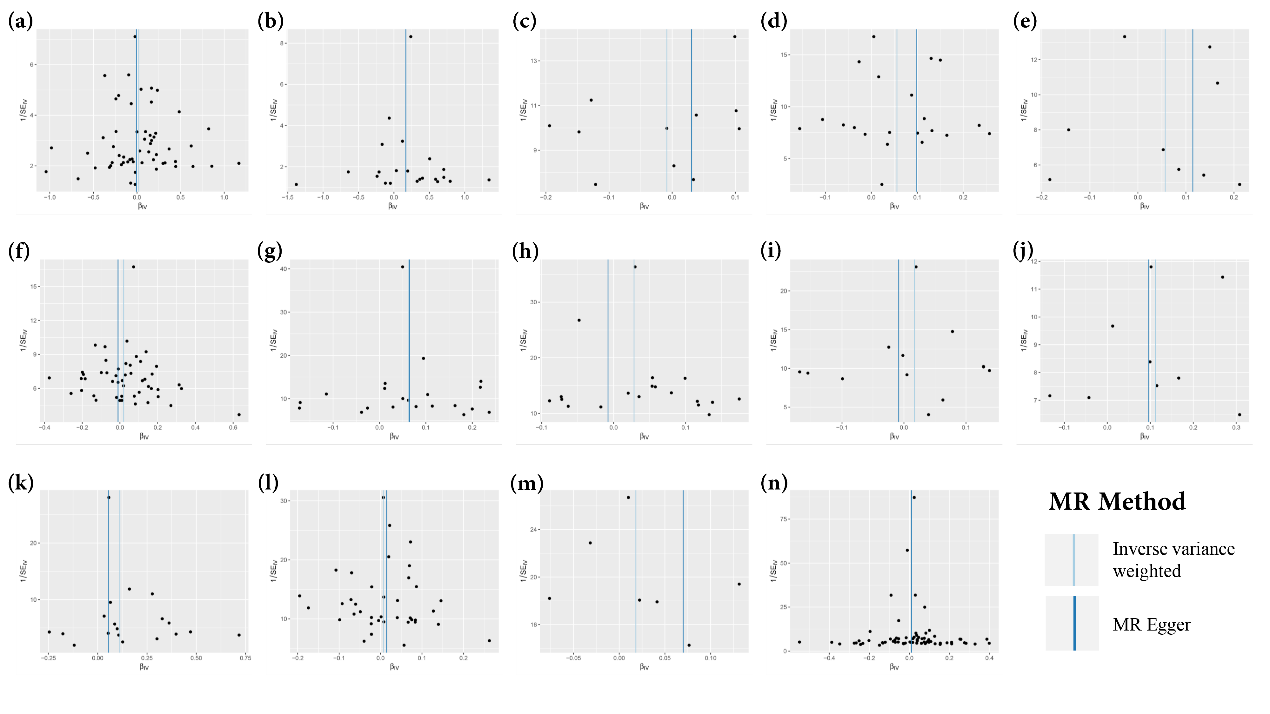


a. Asthma and temporomandibular disorders (TMDs); b. Ankylosing spondylitis (AS) and TMDs; c. Celiac disease and TMDs; d. Graves’ disease and TMDs; e. Hashimoto thyroiditis and TMDs; f. Inflammatory bowel disease (IBD) and TMDs; g. Multiple sclerosis (MS) and TMDs; h. Primary biliary cirrhosis (PBC) and TMDs; i. Primary sclerosing cholangitis (PSC) and TMDs; j. Psoriasis vulgaris and TMDs; k. Rheumatoid arthritis (RA) and TMDs; l. Systemic lupus erythematosus (SLE) and TMDs; m. Sjogren’s syndrome (SS) and TMDs; n. Type 1 diabetes (T1D) and TMDs.

**Supplementary Fig. 3 Leave-one-out sensitivity analysis for 14 autoimmune diseases on temporomandibular disorders.**


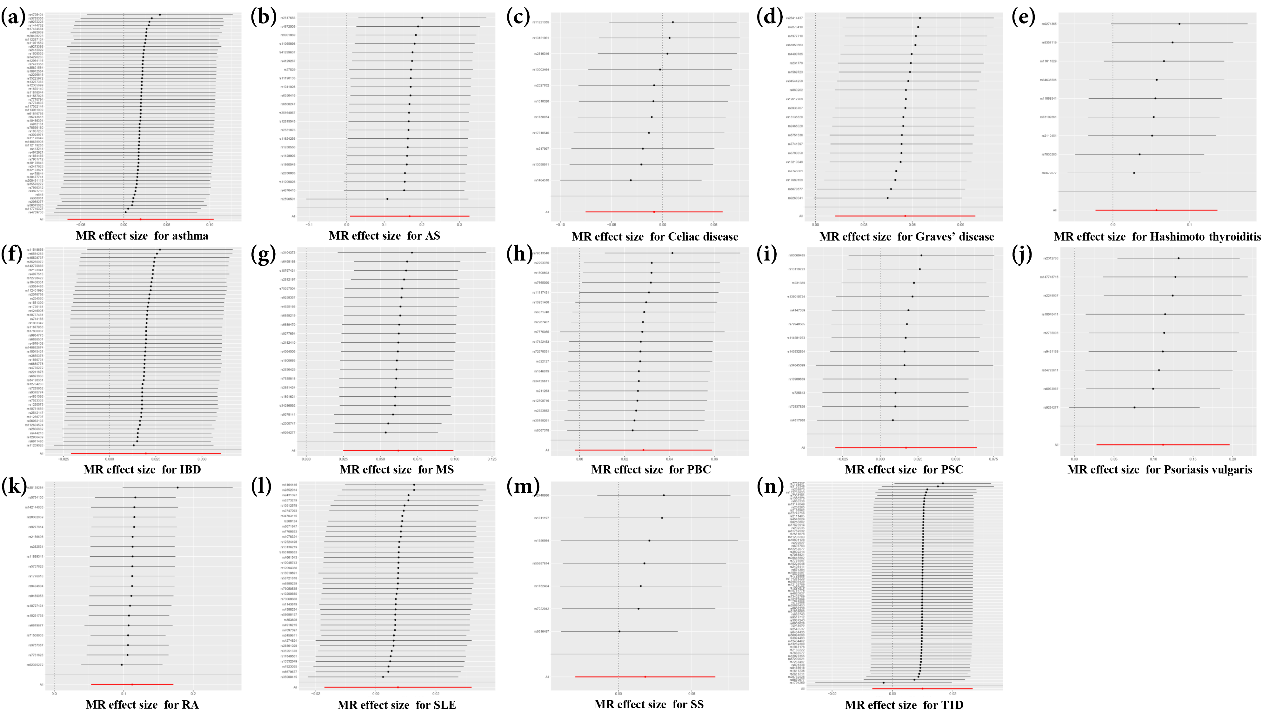


a. Asthma and temporomandibular disorders (TMDs); b. Ankylosing spondylitis (AS) and TMDs; c. Celiac disease and TMDs; d. Graves’ disease and TMDs; e. Hashimoto thyroiditis and TMDs; f. Inflammatory bowel disease (IBD) and TMDs; g. Multiple sclerosis (MS) and TMDs; h. Primary biliary cirrhosis (PBC) and TMDs; i. Primary sclerosing cholangitis (PSC) and TMDs; j. Psoriasis vulgaris and TMDs; k. Rheumatoid arthritis (RA) and TMDs; l. Systemic lupus erythematosus (SLE) and TMDs; m. Sjogren’s syndrome (SS) and TMDs; n. Type 1 diabetes (T1D) and TMDs.
